# Magnitude of Bone Disease in Transfusion-Dependent and Non-Transfusion-Dependent β-Thalassemia Patients

**DOI:** 10.7759/cureus.56012

**Published:** 2024-03-12

**Authors:** Rawand P Shamoon, Ahmed K Yassin, Negar Omar, Muhammad D Saeed, Reving Akram, Naska N Othman

**Affiliations:** 1 Department of Pathology, College of Medicine, Hawler Medical University, Erbil, IRQ; 2 Department of Laboratory Medical Sciences, College of Health Sciences, Catholic University in Erbil, Erbil, IRQ; 3 Department of Hematology, Nanakali Hospital for Blood Diseases and Cancer, Erbil, IRQ; 4 Department of Internal Medicine, College of Medicine, Hawler Medical University, Erbil, IRQ; 5 Department of Physiotherapy and Rehabilitation, Erbil Teaching Hospital, Erbil, IRQ; 6 Department of Hematology, Thalassemia Care Center, Erbil, IRQ; 7 Department of Pediatrics, Thalassemia Care Center, Erbil, IRQ

**Keywords:** non-transfusion dependent thalassemia, transfusion-dependent thalassemia (tdt), bmd z-score, dexa scan, beta-thalassemia

## Abstract

Introduction

β-Thalassemia is a common inherited disease in the northern part of Iraq. A considerable number of transfusion-dependent (TDT) and non-transfusion-dependent (NTDT) β-thalassemia patients suffer bone problems. The objective of this study was to evaluate the degree of bone disease in the TDT and NTDT patients using a dual-energy X-ray absorptiometry (DEXA) scan.

Patients and methods

In this study, 53 TDT and 20 NTDT patients aged ≥10 years were enrolled. Their bone status was assessed using the DEXA scan at the lumbar spine (L1-L4) and femoral neck. The effect of physical, biochemical, and hormonal characteristics on the bone mineral density (BMD) parameters was evaluated. The value of the BMD Z-score was the measure to decide on the magnitude of bone disease.

Results and discussion

The mean age of the enrolled patients was 24.1 years. The BMD Z-score values were significantly lower among the TDT patients at the lumbar spine and femoral neck (BMD Z-score: -2.05 and -1.51 versus -2.29 and -0.71; p=0.044 and 0.009, respectively). The proportion of osteoporosis at the lumbar spine was significantly higher in the TDT group than in the NTDT group (69.8% versus 40%; p <0.001). The BMD Z-score correlated significantly with patient BMI and parathyroid hormone (PTH) level in both the TDT and NTDT groups. No correlation was found with age, hemoglobin (Hb), and serum levels of calcium, vitamin D, ferritin, phosphorus, and alkaline phosphatase (ALP).

Conclusions

Impaired bone density was encountered at high proportions in our thalassemia patients. TDT patients suffered more severe bone disease than NTDT patients.

## Introduction

β-Thalassemia is an inherited autosomal recessive disorder caused by the reduction or absence of β-globin chain synthesis. It presents in one of the three clinical phenotypes: transfusion-dependent thalassemia major (TM), non-transfusion-dependent thalassemia intermedia (NTDT), and thalassemia minor [1]. The diagnosis of transfusion-dependent thalassemia (TDT) is usually made within the first two years of life with patients having significant anemia requiring regular blood transfusion and hepatosplenomegaly. Patients with NTDT are usually diagnosed later with moderate anemia that requires intermittent or no blood transfusion [2].

In recent decades, the life expectancies of thalassemic patients have significantly increased owing to the improved quality of provided services, regular blood transfusion, and effective iron chelation. On the other hand, this has increased the rate of morbidity and complications. Bone diseases, such as low bone density, osteopenia, and osteoporosis, pain, deformities, fractures, and nerve compression at the spine and femoral neck are the major sources of morbidity and long-term complications of TDT and NTDT [3,4]. Osteoporosis is a reduction in bone mass due to increased bone resorption with an increasing risk of fractures [5]. Reduced bone mass in thalassemia is attributed to a variety of reasons, such as increased marrow erythropoiesis, marrow expansion, genetic factors, extensive iron overload, iron chelators, hormonal deficits like hypothyroidism, hypoparathyroidism, and hypogonadism, nutritional and vitamin deficiency, and decreased physical activity [6-11].

Dual X-ray absorptiometry (DEXA) is the most widely used method for assessing bone mineral density (BMD). It uses radiation to measure bone density in certain bone sites, namely the lumbar spine and femoral head. DEXA scan is a relatively safe, quite useful, and convenient tool for assessing bone density [12]. In the northern Iraqi region, β-thalassemia represents a communal medical problem. The carrier rate of β-thalassemia in Erbil, the capital city of the region with a population of 2.25 million, is estimated at 9% [13,14]. Currently, more than 750 TDT and 150 NTDT cases have been documented and registered at the Erbil Thalassemia Care Center. Many thalassemics attending the care center complain of diffuse bone pain and problems in the lower back; a few others have a history of fractures caused by irrelevant force or trauma. No local data are available regarding the magnitude of bone disease in thalassemia; therefore, we deemed it necessary to include a group of our TDT and NTDT patients in a study to scrutinize the extent of bone disease by measuring the BMD and correlating it to the patients’ clinical data and biochemical and hormonal assays.

## Materials and methods

This cross-sectional comparative study included 53 TDT and 20 NTDT patients. It was carried out at the Thalassemia Care Center of Erbil City, northern Iraq, from September 2022 to April 2023. Patients aged 10 years and above were conveniently enrolled in this study while visiting the care center. Informed written consent was obtained from the included patients or their guardians; the study was approved by the ethical committee of Hawler Medical University. Patients <10 years and those who had bone marrow transplantation were not included. Patients receiving drugs affecting BMD, such as antiepileptic drugs, oral calcium, vitamin D, and corticosteroids, were also excluded.

Sociodemographic and clinical data were recorded during the patients’ regular visits to the care center. After clinical examination, body mass index (BMI) was calculated. Histories of transfusion and chelation therapy, splenectomy, bone pain, and fractures were specifically scrutinized. Pretransfusion hemoglobin (Hb) was recorded. Levels of calcium, vitamin D, ferritin, phosphorus, alkaline phosphatase (ALP), and parathyroid hormone (PTH) were measured using an automated analyzer (Cobas 6000 modular system, Roche Germany). BMD was assessed using a Hologic QDR DEXA scan device to evaluate the BMD and bone mineral concentration (BMC) at the lumbar spine (L1-L4) and the femoral neck. DEXA scan was performed at the Physiotherapy and Rehabilitation Centre of Erbil Teaching Hospital. After performing the DEXA scan, the Z-score was automatically generated at the two measuring sites, the lumbar spine and femoral neck. As per the WHO criteria for the diagnosis of osteoporosis, a BMD Z-score of ≥-1 was considered normal, a Z-score between -1 and -2.5 was considered osteopenia, and a Z-score of ≤-2.5 was considered osteoporosis. The BMD Z-score was correlated with the patients’ clinical and laboratory results.

Statistical analysis was performed using the Statistical Package for Social Sciences (SPSS, version 25). Chi-square test and Fisher’s exact test were used to compare proportions. Student’s t-test of two independent samples was used to compare the means of the two samples. Analysis of variance (ANOVA) was employed to compare more than two means. A p-value of ≤0.05 was considered statistically significant.

## Results

The BMD of 53 TDT and 20 NTDT patients was evaluated in this study. Their ages ranged between 10 and 59 years with a mean of 24.1 ± 11.8 years. They included 47 (64.4%) males and 26 (35.6%) females. The mean patient age and the mean age at diagnosis of the TDT group were significantly lower than those of the NTDT group (21.3 and 0.7 years versus 31.4 and 19.3 years, respectively). The rate of splenectomy was higher among the TDT group. The mean BMD Z-score values at both measuring sites were significantly lower in the TDT patients than in the NTDT group. The demographic, clinical, and laboratory parameters of TDT and NTDT patients are illustrated in Table 1.

**Table 1:** Characteristics of TDT and NTDT patients TDT: transfusion-dependent β-thalassemia; NTDT: non-transfusion-dependent β-thalassemia; BMI: body mass index; Hb: hemoglobin; ALP: alkaline phosphatase; PTH: parathyroid hormone; BMD: bone mineral density. *Student’s t-test. †Chi-square test.

Characteristics	TDT	NTDT	p-value
Age (year) (mean ± SD (range))	21.3 ± 7.8 (11, 41)	31.4 ± 16.8 (10, 58)	0.017^*^
Gender			0.087^†^
Male (No. (%))	31 (58.5)	16 (80.0)	
Female (No. (%))	22 (41.5)	4 (20.0)	
Age of diagnosis (year) (mean ± SD (range))	0.7 ± 0.6 (0.2, 3)	19.3 ± 14.5 (2, 44)	<0.001^*^
BMI (kg/m^2^) (mean ± SD (range))	20.8 ± 3.1 (14.2, 28)	21.7 ± 5.2 (14.2, 28.0)	0.442^*^
Splenectomy			0.017^†^
Yes (No. (%))	27 (50.9)	4 (20.0)	
No (No. (%))	26 (49.1)	16 (80.0)	
Hb (g/dL) (mean ± SD (range))	8.5 ± 0.9 (6.4, 10.9)	10.0 ± 1.5 (8.30, 14.0)	<0.001^*^
Calcium (mg/dL) (mean ± SD (range))	9.5 ± 0.8 (6.8, 11.0)	10.2 – 1.0 (8.20, 12.28)	0.003^*^
Vitamin D (ng/mL) (mean ± SD (range))	18.6 ± 6.8 (4.0, 33.7)	23.1 ± 13.6 (8.68, 67.50)	0.170^*^
Ferritin (ng/mL) (mean ± SD (range))	3701.2 ± 2737.6 (431.0, 11880.0)	1272.3 ± 1104.8 (24.40, 3463.00)	<0.001^*^
Phosphorus (mg/dL) (mean ± SD (range))	4.7 ± 0.9 (2.3, 7.6)	4.9 ± 0.9 (3.10, 6.54)	0.532^*^
ALP (U/L) (mean ± SD (range))	162.9 ± 55.8 (61.6, 304.0)	130.5 ± 45.2 (73.00, 215.0)	0.023^*^
PTH (pg/mL) mean ± SD (range)	5.6 ± 4.0 (1.2, 17.4)	20.7 ± 11.4 (1.65, 39.99)	<0.001^*^
BMD Z-score, lumbar spine (mean ± SD (range))	-2.95 ± 1.07 (-5.20, - 0.80)	-2.29 ± 1.57 (-5.00, - 1.30)	0.044^*^
BMD Z-score, femoral neck (mean ± SD (range))	-1.51 ± 1.02 (-4.10, - 1.30)	-0.71 ± 1.36 (-2.90, - 1.70)	0.009^*^

Osteoporosis and osteopenia were encountered at significantly higher rates among the TDT patients (p<0.001). The rate of osteoporosis was higher in the lumbar spine than in the femoral neck (Table 2).

**Table 2:** Prevalence of osteoporosis and osteopenia in TDT and NTDT patients TDT: transfusion-dependent β-thalassemia; NTDT: non-transfusion-dependent β-thalassemia; BMD: bone mineral density; BMI: body mass index. *Chi-square test.

	TDT (No. (%))	NTDT (No. (%))	p-value
BMD Z-score spine (L1-L4)			
Osteoporosis	37 (69.8)	8 (40.0)	<0.001^*^
Osteopenia	13 (24.5)	9 (45.0)	
Normal	3 (5.7)	3 (15.0)	
BMD Z-score femoral neck			
Osteoporosis	7 (13.2)	2 (10.0)	<0.001^*^
Osteopenia	30 (56.6)	6 (30.0)	
Normal	16 (30.2)	12 (60.0)	
Total	53 (100.0)	20 (100.0)	

A history of fracture was encountered in 10 (18.9%) TDT patients and one (5%) NTDT patient. The mean age of the TDT patients with a history of fracture was significantly greater than that of patients with no history of fracture (27.8 years versus 20.8 years, p=0.002). The mean BMD Z-score of patients with a history of fracture was lower than that of patients with no history of fracture; however, the difference did not reach statistical significance (p=0.19). All clinical and laboratory parameters, including splenectomy and PTH level, did not show any significant relation with a history of fractures.

The degree of bone disease, represented by the BMD Z-score, was correlated with the patients’ clinical parameters as illustrated in Tables 3, 4. Among the TDT patients, only the PTH level was significantly correlated with the rate of osteoporosis. The remaining parameters did not show any correlation except BMI which significantly varied with the BMD Z-score at the lumbar spine. In the NTDT patient group, no significant correlation was detected between the studied parameters and bone disease.

**Table 3:** Relation between TDT patient’s characteristics and bone disease TDT: transfusion-dependent β-thalassemia; BMD: bone mineral density; BMI: body mass index; Hb: hemoglobin; ALP: alkaline phosphatase; PTH: parathyroid hormone. *Analysis of variance (ANOVA) test. †Fisher Exact test.

Overall	BMD Z score	Spine	p-value	Neck femur	p-value
Age (yr) (mean ± SD)	Osteoporosis	20.19 ± 8.40	0.283^*^	19.29 ± 6.95	0.600^*^
	Osteopenia	23.85 ± 6.20		22.23 ± 8.59	
	Normal	24.33 ± 4.62		20.50 ± 6.88	
Males (No. (%))	Osteoporosis	23 (74.2)	0.636^†^	3 (9.7)	0.486^†^
	Osteopenia	7 (22.6)		17 (54.8)	
	Normal	1 (3.2)		11(35.5)	
Females (No. (%))	Osteoporosis	14 (63.6)		4 (18.2)	
	Osteopenia	6 (27.3)		13 (59.1)	
	Normal	2 (9.1)		5 (22.7)	
BMI (kg/m^2^) (mean ± SD)	Osteoporosis	19.77 ± 2.57	0.001^*^	19.15 ± 3.59	0.295^*^
	Osteopenia	22.97 ± 2.83		20.79 ± 3.23	
	Normal	23.19 ± 5.39		21.38 ± 2.65	
Age at diagnosis (yr) (mean ± SD)	Osteoporosis	0.70 ± 0.53	0.485^*^	0.49 ± 0.45	0.338^*^
	Osteopenia	0.78 ± 0.73		0.83 ± 0.65	
	Normal	1.12 ± 0.44		0.69 ± 0.46	
Hb (g/dL) (mean ± SD)	Osteoporosis	8.30 ± 0.83	0.068^*^	8.56 ± 0.39	0.430^*^
	Osteopenia	8.82 ± 0.91		8.58 ± 0.94	
	Normal	9.13 ± 0.45		8.24 ± 0.87	
Calcium (mg/dL) (mean ± SD)	Osteoporosis	9.60 ± 0.72	0.088^*^	9.43 ± 0.62	0.585^*^
	Osteopenia	9.06 ± 0.80		9.39 ± 0.89	
	Normal	9.57 ± 0.68		9.63 ± 0.56	
Vitamin D (ng/mL) (mean ± SD)	Osteoporosis	17.97 ± 7.31	0.529^*^	21.16 ± 0.76	0.158^*^
	Osteopenia	19.50 ± 5.56		17.02 ± 9.85	
	Normal	22.00 ± 3.61		20.35 ± 6.93	
Ferritin (ng/mL) (mean ± SD)	Osteoporosis	4014.62 ± 2677.98	0.227^*^	5249.43 ± 2062.21	0.268^*^
	Osteopenia	3363.15 ± 2998.07		3553.97 ± 3010.94	
	Normal	1300.33 ± 798.14		3299.88 ± 2329.14	
Phosphorous (mg/dL) (mean ± SD)	Osteoporosis	4.73 ± 0.73	0.975^*^	4.57 ± 0.81	0.975^*^
	Osteopenia	4.68 ± 1.26		4.74 ± 0.95	
	Normal	4.63 ± 0.32		4.71 ± 0.86	
ALP (mg/dL) (mean ± SD)	Osteoporosis	165.12 ± 54.69	0.305^*^	169.79 ± 43.14	0.683
	Osteopenia	167.59 ± 61.72		166.69 ± 57.28	
	Normal	114.47 ± 22.81		152.64 ± 59.54	
PTH (pg/mL) (mean ± SD)	Osteoporosis	4.70 ± 3.61	0.029^*^	2.20 ± 1.65	0.035^*^
	Osteopenia	8.05 ± 4.40		5.81 ± 3.57	
	Normal	6.53 ± 3.39		6.79 ± 4.80	

**Table 4:** Relation between NTDT patient’s characteristics and bone disease NTDT: non-transfusion-dependent β-thalassemia; BMD: bone mineral density; BMI: body mass index; Hb: hemoglobin; ALP: alkaline phosphatase; PTH: parathyroid hormone. *Analysis of variance (ANOVA) test. †Fisher Exact test.

Overall	BMD Z score	Spine	p-value	Neck femur	p-value
Age (yr) (mean ± SD)	Osteoporosis	24.75 ± 9.06	0.122^*^	34.00 ± 33.94	0.795^*^
	Osteopenia	31.78 ± 12.41		27.33 ± 19.99	
	Normal	48.00 ± 14.73		33.00 ±13.81	
Males (No. (%))	Osteoporosis	6 (37.5)	0.614^†^	0 (0.0)	0.006^†^
	Osteopenia	8 (50.0)		4 (25.0)	
	Normal	2 (12.5)		12 (75.0)	
Females (No. (%))	Osteoporosis	2 (50.0)		2 (50.0)	
	Osteopenia	1 (25.0)		2 (50.0)	
	Normal	1 (25.0)		0 (0.0)	
BMI (kg/m^2^) (mean ± SD)	Osteoporosis	18.35 ± 2.74	0.003^*^	20.41 ± 2.97	0.452^*^
	Osteopenia	22.26 ± 4.93		19.71 ± 6.10	
	Normal	29.03 ± 1.90		22.93 ± 4.93	
Age at diagnosis (yr) (mean ± SD)	Osteoporosis	13.00 ± 14.90	0.233^*^	20.00 ± 22.63	0.651^*^
	Osteopenia	21.67 ± 15.06		14.50 ± 15.41	
	Normal	28.67 ± 1.53		21.50 ± 13.84	
Hb (g/dL) (mean ± SD)	Osteoporosis	9.20 ± 0.59	0.060^*^	9.15 ± 0.21	0.093^*^
	Osteopenia	10.83 ± 1.77		9.12 ± 0.78	
	Normal	9.63 ± 1.15		10.58 ± 1.61	
Calcium (mg/dL) (mean ± SD)	Osteoporosis	972.53 ± 709.33	0.192^*^	1091.50 ± 267.99	0.958^*^
	Osteopenia	1755.53 ± 1379.12		1222.60 ± 1172.08	
	Normal	622.17 ± 426.91		1327.33 ± 1211.08	
Vitamin D (ng/mL) (mean ± SD)	Osteoporosis	10.65 ± 1.18	0.200^*^	9.75 ± 0.49	0.184^*^
	Osteopenia	9.79 ± 0.89		10.79 ± 1.01	
	Normal	9.91 ± 0.10		9.90 ± 0.98	
Ferritin (ng/mL) (mean ± SD)	Osteoporosis	1096.9 ± 617.6	0.19^*^	1091.5 ± 267.9	0.98^*^
	Osteopenia	1755.3 ± 1379.1		1222.6 ± 1172.1	
	Normal	622.2 ± 426.9		1327.3 ± 1211.1	
Phosphorous (mg/dL) (mean ± SD)	Osteoporosis	4.45 ± 1.11	0.282^*^	3.80 ± 0.99	0.222^*^
	Osteopenia	5.10 ± 0.67		4.88 ± 0.88	
	Normal	5.18 ± 0.84		5.02 ± 0.87	
ALP (mg/dL) (mean ± SD)	Osteoporosis	153.38 ± 48.36	0.145^*^	155.00 ± 84.85	0.728^*^
	Osteopenia	120.11 ± 42.14		130.83 ± 56.79	
	Normal	100.33 ± 14.22		126.17 ± 35.83	
PTH (pg/mL) (mean ± SD)	Osteoporosis	16.08 ± 12.28	0.242^*^	13.72 ± 17.07	0.576^*^
	Osteopenia	25.41 ± 11.26		19.15 ± 11.27	
	Normal	18.81 ± 3.98		22.62 ± 11.29	

## Discussion

In thalassemia, osteoporosis and osteopenia are known complications that are observed even in well-treated patients. Bone disease in thalassemia involves complex interactions of various factors affecting the growing bones. Manifestations of defective BMD in β-thalassemia are still not very well understood despite the progress that has been made in the understanding of the natural history and pathogenesis of the disease [11]. The current study showed significant bone disease among both TDT and NTDT patients. The DEXA scan device is programmed to calculate BMD scores. The BMD Z-score compares one’s bone density to the average value for a person of the same age and gender. The BMD T-score is a standard deviation that calculates how much a result varies from the average. The latter is recommended to be used for adults. In our cohort, the majority of the enrolled were children and adolescents; therefore, we adopted the BMD Z-score as the main parameter of bone density. In this study, we did not include children below 10 years for two reasons: first, we seldom receive complaints of bone problems and backaches from thalassemic children below 10 years; second, it is not easy to perform the DEXA scan on small children because of difficulty of proper positioning by the technicians.

In this study, the mean BMD Z-score values of the TDT patients were significantly lower than those of the NTDT patients. The differences in the BMD Z-scores were significant at the two scanned sites, the lumbar spine and femoral neck. Studies on thalassemics patients in Iran showed more severe bone disease among TDT patients in comparison to NTDT patients; however, the differences in the BMD Z-core values did not reach a statistically significant level [15,16]. Osteoporosis and osteopenia were recorded at significantly higher proportions among the TDT patients in comparison to the NTDT group. Out of 53 enrolled TDT patients, 50 (94.3%) had defective BMD Z-scores at the lumbar spine, while 37 (69.8%) had abnormal Z-scores at the femoral neck. On the other hand, the rates of defective Z-score were lower among the 20 NTDT patients, 85% and 40% at the lumbar spine and femoral neck, respectively. Many studies have reported higher rates of osteoporosis among TDT than NTDT patients [17,18]. Osteoporosis was recorded at a higher rate in the lumbar spine than in the femoral neck. This pattern of bone disease in thalassemia is expected and has been repeatedly reported in previous studies [19,20]. The explanation of this differential bone mineral loss is attributed to the fact that the lumbar spine consists of trabecular bone and wide marrow space, and the accelerated hematopoiesis and progressive bone expansion, which is part of thalassemia pathology, affects the spine more severely than the proximal femur.

It is well known that pathological changes in thalassemia have cumulative effects, and therefore, complications become more apparent with age. We did not find a significant relationship between the BMD Z-score and patient age. Many studies reported similar findings [18,21]; however, others found a significant relation with age [9,22]. A possible reason for this discrepancy is that, unlike other studies, we did not include patients younger than 10 years. The history of fractures varied significantly with the patient age. In our cohort, 11 patients had a history of limb fractures caused by irrelevant force or trauma; of them, 10 were TDT patients. Patients with a history of fracture had more advanced bone resorption with a lower BMD Z-score compared to those with no history of fracture. The PTH level as well as the other clinical and laboratory parameters did not show any relation with fractures. Retarded growth is a complication in patients with thalassemia. In this cohort, there was no significant difference in BMI between the TDT and NTDT patients; however, spinal osteoporosis correlated significantly with low BMI in both groups. This finding is consistent with many previous studies which concluded that low BMI is a significant predictor of impaired BMD [21,23].

As expected, significant variations were detected in most of the hematological and biochemical parameters between the TDT and NTDT patients including the Hb and serum levels of ferritin, calcium, ALP, and PTH. However, all the studied parameters, except the PTH level in the TDT group, did not show any correlation with the BMD Z-score. It has been reported that normalization of Hb does not affect unbalanced bone turnover in thalassemic patients [22,24]. The mean serum levels of vitamin D were low in the two studied cohorts. However, vitamin D levels did not reveal significant correlations with BMD Z-score values. The same findings were reported in many previous studies [17,24]. Low vitamin D level in thalassemia is mainly attributed to high ferritin levels which in turn affect the hydroxylation of vitamin D in the liver. This is partly due to decreased physical activity. Some studies have shown that regular intake of calcium and vitamin D can be important for bone formation and preventing osteoporosis [25,26]. Increased levels of ALP in thalassemia are multifactorial; renal failure, hyperthyroidism, high intake of phosphate, and hypoparathyroidism are possible contributors. No significant correlation between BMD Z-score values and levels of ALP and phosphorus was detected in the patients at either scanned site. These findings are comparable to those of Izadyar et al [27], although Ansari-Moghadam et al. found a significant difference between the BMD Z-score and phosphorous level in their TDT cohort [18]. It is worth mentioning here that non-enrolling children younger than 10 years and lack of few important biomarkers of bone metabolism, such as C-terminal telopeptide of type-1 collagen (CTX-1) and osteocalcin (OC), that we could not measure their levels in our patients because of limited financial resources, represent the main limitations of our work. However, we believe that, in this developing part of the world, our results will be very useful for practitioners and care providers and will raise their awareness about bone disease among thalassemic patients.

## Conclusions

In conclusion, a significant rate of bone disease was detected in the enrolled thalassemic patients. Osteoporosis was encountered significantly more often in TDT patients. Impaired BMD correlated significantly with patient BMI and PTH levels. The rates of osteoporosis and osteopenia did not vary with the patient age. No significant relationships between calcium, vitamin D, phosphorus, and ALP and bone disease were noted.
